# In Vivo Versus In Vitro Somatostatin Receptor Expression in Neuroendocrine Neoplasms: A Systematic Review and Meta-Analysis of Correlation Studies

**DOI:** 10.3390/ijms26146551

**Published:** 2025-07-08

**Authors:** Elisabetta Perrone, Giorgio Treglia, Romina Grazia Giancipoli, Lucia Leccisotti, Guido Rindi, Vittoria Rufini

**Affiliations:** 1 Nuclear Medicine Unit, Department of Diagnostic Imaging, Oncological Radiotherapy and Hematology, Fondazione Policlinico Universitario A. Gemelli IRCCS, 00168 Rome, Italy; elisabetta.perrone@guest.policlinicogemelli.it (E.P.); lucia.leccisotti@policlinicogemelli.it (L.L.); vittoria.rufini@policlinicogemelli.it (V.R.); 2 Faculty of Biomedical Sciences, Università della Svizzera Italiana (USI), 6900 Lugano, Switzerland; 3 Division of Nuclear Medicine, Imaging Institute of Southern Switzerland, Ente Ospedaliero Cantonale, 6500 Bellinzona, Switzerland; 4 Department of Nuclear Medicine and Molecular Imaging, Lausanne University Hospital, University of Lausanne, 1015 Lausanne, Switzerland; 5 PET-CT Center, Fondazione Policlinico Universitario A. Gemelli IRCCS, 00168 Rome, Italy; romina.giancipoli@guest.policlinicogemelli.it; 6 European Neuro-Endocrine Tumor Society (ENETS) Center of Excellence, Fondazione Policlinico Universitario A. Gemelli IRCCS, Università Cattolica del Sacro Cuore, 00168 Rome, Italy; guido.rindi@policlinicogemelli.it; 7 Section of Nuclear Medicine, Department of Radiological Sciences and Hematology, Università Cattolica del Sacro Cuore, 00168 Rome, Italy; 8 Section of Anatomic Pathology, Department of Life Sciences and Public Health, Università Cattolica del Sacro Cuore, 00168 Rome, Italy; 9 Unit of Head and Neck, Thoracic and Endocrine Pathology, Department of Woman and Child Health Sciences and Public Health, Fondazione Policlinico Universitario A. Gemelli IRCCS, 00168 Rome, Italy

**Keywords:** neuroendocrine neoplasms, PET/CT, immunohistochemistry, somatostatin receptors, somatostatin analogs, nuclear medicine, meta-analysis

## Abstract

Well-differentiated neuroendocrine neoplasms (NENs) are characterized by hyperexpression on the cell membrane of somatostatin receptors (SSTRs). The demonstration of SSTRs, mainly the subtype 2 (SSTR2), is the prerequisite for diagnostic and therapeutic strategies with radiolabeled somatostatin analogs (SSAs). SSTRs can be routinely demonstrated in vivo by SSA-positron emission tomography/computed tomography (SSA-PET/CT) and in vitro by immunohistochemistry (IHC). This systematic review and meta-analysis aimed to gather evidence from the available literature on the correlation between the in vivo PET/CT and in vitro IHC SSTR expression in NEN patients. A systematic review and meta-analysis were conducted following the preferred reporting items for systematic reviews and meta-analyses (PRISMA) 2020 guidelines. A comprehensive literature search was performed in PubMed/MEDLINE and Cochrane Library, selecting studies correlating SSTR expression in NENs via IHC and SSA-PET/CT. Data extraction, quality assessment, and statistical analysis were performed. Eleven studies met the inclusion criteria for systematic review (345 patients). Of these, eight studies (299 patients) provided sufficient quantitative data for meta-analysis. The pooled concordance between SSA-PET/CT and IHC was 76% (95% CI: 67.7–84.2), indicating a good correlation between in vivo and in vitro SSTR2 expression. Heterogeneity among studies was moderate (I^2^ = 65%), reflecting different patient cohorts and methodologies regarding both SSA-PET/CT and IHC. No significant publication bias was detected. Our results confirmed good agreement between in vivo tumor uptake with SSA-PET/CT and in vitro SSTR2 expression with IHC, highlighting the potential of using IHC for clinical decision-making in NEN patients when SSA-PET/CT is not available.

## 1. Introduction

Somatostatin receptors (SSTRs) are membrane proteins that bind to somatostatin and its analogs; upon activation by ligand binding, SSTRs control the secretion of several hormones. SSTRs include five subtypes (SSTR1–5) and all are conventional G protein-coupled receptors that possess a seven transmembrane α-helix structure; the extracellular region is involved in ligand binding, and the intracellular region couples with and activates G proteins [1]. Among SSTRs, SSTR2 is the most widely expressed; SSTRs are identified in several organs and their physiological functions vary depending on the tissues and cells in which they are present [1]. In addition, SSTRs are expressed in various neoplasms and can serve as a biomarker for the diagnosis and treatment of several diseases, such as neuroendocrine neoplasms (NENs) and meningiomas [1].

NENs are heterogeneous tumors originating from neuroendocrine cells and can occur anywhere in the body. NENs comprise a broad family of tumors based on their location and tumor grade, with increasing incidence worldwide. Some NENs are characterized by hormonal hypersecretion causing functional abnormalities and related symptoms, whereas others are non-functional tumors [2].

The distinctive characteristic of well-differentiated NENs—as such defined neuroendocrine tumors (NETs)—is the hyperexpression on the cell membrane of SSTRs, which act as key regulators of cell proliferation, protein synthesis, and hormone secretion in neuroendocrine cells [1].

The demonstration of SSTRs, mainly SSTR2, is the prerequisite for diagnostic and therapeutic strategies with radiolabeled somatostatin analogs (SSAs), an approach defined as theranostics [2]. Indeed, since the approval of the SSTR agonist Lu-177 DOTATATE (Lutathera^®^) [3] this radiopharmaceutical has become part of the therapeutic arsenal for patients with gastro-entero-pancreatic (GEP) NETs after conventional therapy, and there is evidence that it is potentially applicable in the first-line setting for inoperable grade 2 or 3 GEP NETs [4,5]. For imaging, positron emission tomography/computed tomography (PET/CT) with SSAs labeled with Ga-68 or Cu-64 has replaced In-111 pentetreotide scintigraphy due to higher spatial resolution and consequent higher sensitivity in lesion detection, lower radiation dose, and shorter study duration. Indeed, radiolabeled SSAs are routinely used for in vivo SSTR characterization, disease staging, and therapeutic monitoring. In addition, based on the intensity of radioligand uptake, patients suitable for Lu-177 DOTATATE are selected [6].

Besides in vivo characterization, SSTRs can be identified in vitro by reverse transcriptase-polymerase chain reaction (RT-PCR) on tumor tissue nucleic acid extracts, or on tissue by autoradiography or immunohistochemistry (IHC); the latter technique is a sensitive, unexpensive, and reproducible method with widespread access, which identifies SSTR subtypes on formalin-fixed, paraffin-embedded tumor samples, a material usually available after diagnostic procedures [7,8,9].

The correlation between in vivo expression by imaging and SSTR IHC staining was initially assessed by In-111 pentetreotide scintigraphy in patients with NETs of different origins [10,11,12]. Subsequent studies explored the correlation between in vivo studies with the more sensitive PET/CT and in vitro IHC SSTRs analysis [13,14,15,16,17,18,19,20,21,22,23].

Even if previous studies have already evaluated the correlation between the in vivo and in vitro expression of SSTRs in NEN patients, a systematic review and a meta-analysis on this topic could offer several advantages over single studies, increasing the statistical power, improving the precision of effect estimates, and providing a more comprehensive overview about this correlation. By pooling data from multiple studies, this meta-analysis could identify trends and patterns that might be missed in individual investigations, especially when dealing with small sample sizes or conflicting results [24,25,26].

The aim of this systematic review and meta-analysis is to gather evidence of the available literature on the correlation between the in vivo (SSA-PET/CT) and in vitro (IHC) expression of SSTRs in NEN patients, summarizing available results and highlighting clinical implications on radioligand imaging and therapy. The results of the meta-analysis could have important clinical implications, as if a significant correlation between the in vivo and in vitro expression of SSTRs in NEN patients is reported then this could be the rationale for using IHC as a surrogate of SSA-PET/CT when this imaging technique is not available, or for using PET imaging able to detect SSTR-positive lesions for the selection of patients for radioligand therapy.

## 2. Materials and Methods

### 2.1. Protocol, Review Question and Inclusion Criteria

The present systematic review and meta-analysis were performed based on a preset protocol referring to the “Preferred Reporting Items for a Systematic Review and Meta-Analysis” (PRISMA 2020 statement) [24]. PRISMA guidelines were followed for systematic review reporting [24]. There was no previous registration of the study protocol, as the protocols registration is suggested but not mandatory according to PRISMA [24].

As a first step, a concise review question was formulated: “Is there a correlation between the immunohistochemical expression of SSTR in NEN samples and the SSTR in vivo expression at SSA-PET/CT performed in NEN patients?” Following the “Population, Intervention, Comparator, Outcomes” (PICO) framework criteria for study inclusion, only studies exploring the topic of interest were selected. No language or time restrictions were applied. Exclusion criteria included studies on different topics; case reports or briefcase series in the field of interest; and non-original articles in the field of interest like reviews, editorials, comments, or letters. The primary outcome was assessing the correlation between the SSTR expression in NEN samples via IHC and in vivo via SSA-PET/CT. Two authors (E.P. and G.T.) independently carried out the literature search, study selection, quality assessment, and data extraction.

### 2.2. Strategy for Literature Research, Study Selection, and Data Collection and Extraction

Following the establishment of the review questions, the authors performed a comprehensive literature search employing two electronic bibliographic databases (PubMed/MEDLINE and Cochrane Library) to extract only relevant original articles about the topic of interest. The literature research was last updated on July 4, 2024. We used a search algorithm based on the combination of the following terms: (“PET” OR “positron”) AND (“somatostatin”) AND (“immunohisto*”) AND (“neuroendocrine” OR “NET” OR “NEN” OR “carcinoid*”). Moreover, the references of the included studies were also screened to search for additional eligible articles meeting the inclusion criteria. Two authors (E.P and G.T.) independently reviewed the titles and abstracts of the selected studies according to the predetermined inclusion criteria and read the full manuscripts. Disagreements were resolved by consulting a third reviewer (V.R.). Data from all of the included studies were extracted and collected from the two authors independently to avoid potential biases, concerning general study information (first author, year of publication, country, journal, study design, and funding sources), patients’ characteristics (sample size, gender, age, NEN site, and specimen origin—when available) and technical variables both for PET/CT and for IHC (the type of hybrid imaging, the tomograph, the administered radiopharmaceuticals and their activity, the uptake time between injection and image acquisition, the image analysis method, the semiquantitative parameters employed, the IHC targets, the IHC antibodies, and the IHC assessment scores). Thereafter, data regarding the primary outcome (the correlation in percentage between IHC and PET/CT expression of SSTRs in patients with NEN) were extracted and collected, or they were calculated by the reviewers when they were not explicitly provided by the authors.

### 2.3. Quality Assessment (Risk of Bias Assessment)

The QUADAS-2 online tool was employed to assess the quality of the selected articles and to analyze the risk of bias and their pertinence to the review question [25]. Four domains (patient selection, index test, reference standard, flow, and timing) were considered for risk of bias. Three aspects (patient selection, index test, and reference standard) were analyzed to assess the applicability of the included studies [25].

The first part of each domain concerns bias and comprises the following three sections: information used to support the judgment of risk of bias, signaling questions, and judgment of risk of bias. Signaling questions provided by the QUADAS-2 tool flag aspects of study design related to the potential for bias and aim to help reviewers judge the risk of bias for each domain. Signaling questions are answered as “yes,” “no,” or “unclear” and are phrased such that “yes” indicates a low risk of bias. Risk of bias is then judged as “low,” “high,” or “unclear” for each assessed domain. If the answers to all signaling questions for a domain are “yes,” then risk of bias can be judged as low. If any signaling question is answered as “no,” potential for bias exists. Applicability sections are structured in a way similar to that of the bias sections but do not include signaling questions. Concerns about applicability are rated as “low,” “high,” or “unclear” for each assessed domain [25].

Two authors (G.T. and E.P.) independently evaluated the quality of the studies included in this systematic review and meta-analysis.

### 2.4. Statistical Analysis

We calculated through a proportion lesion-based meta-analysis the percentage of concordance among SSA-PET/CT and SSTR expression in IHC. This meta-analysis was performed using a random-effects model which considers the variability between studies. Pooled data were presented with their respective 95% confidence interval (95% CI) values and displayed using a forest plot. Heterogeneity was estimated through the I-square (I^2^) test. The publication bias was assessed through the Egger’s test and visual analysis of the funnel plot. Subgroup analyses were planned only in cases of significant statistical heterogeneity [26]. The statistical software OpenMeta[Analyst] (version 1.0) was used for the meta-analysis.

## 3. Results

### 3.1. Literature Search, Study Characteristics, and Qualitative Synthesis (or Systematic Review)

**Figure 1:**
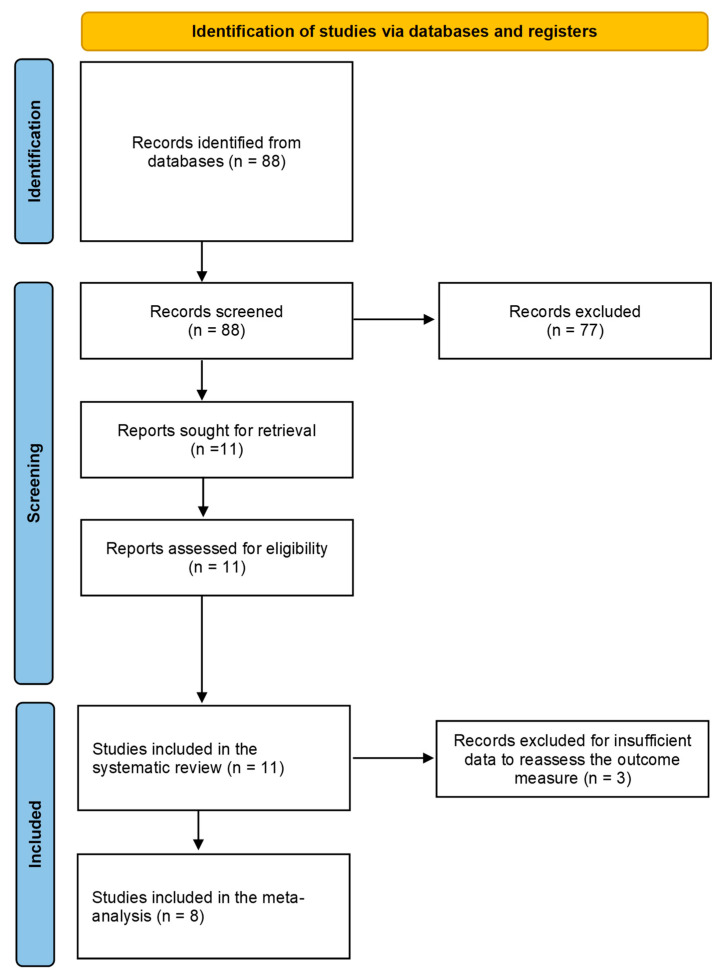
Overview of the literature search process (PRISMA flowchart).

After the literature search conducted across the selected databases, 88 studies were identified. Following the reading of the titles and abstracts, 77 articles were excluded (*n* = 76 being not in the field of interest; *n* = 1 being a preclinical study). The remaining eleven studies were assessed as suitable for inclusion in the systematic review (qualitative synthesis) [13,14,15,16,17,18,19,20,21,22,23]; three of them were excluded due to insufficient data to reassess the outcome measure, leaving eight studies eligible for the meta-analysis (quantitative synthesis) [13,17,18,19,20,21,22,23]. No additional manuscripts were identified after revising the references of the selected records. Figure 1 provides an overview of the study selection process.

The eleven studies meeting the criteria for inclusion in the systematic review (qualitative analysis) are thoroughly analyzed in Table 1 and Table 2. The selected studies were published from 2009 to 2022, mainly in Germany (7/11). Most studies were retrospective, except one study with a prospective design [19]. A total of 345 patients with NENs were included. Tumor distribution among the total of patients included 211 patients with GEP NEN and 40 patients with specified NEN of the pancreas, 71 patients with lung NEN, and 23 patients with NEN of other primary sites. When the data were available, 29 high-grade (G3) tumor lesions were found in 258 patients (11.2%); one study did not specify the number of high-grade patients [15] and two studies included only patients with low-grade tumors [18,22]. Several studies did not specify whether the NEN specimens analyzed with IHC were obtained through biopsy or surgery. Of the remaining specimens, 130 were obtained via biopsy and 151 were collected after surgery. Overall, information on tumor grade and sample type are heterogeneously reported in the included studies.

All articles employed PET/CT as a hybrid imaging device. The peptide administered among the studies was exclusively DOTATOC in two studies [20,21], DOTATATE in two studies [13,23] and DOTANOC in four studies [15,16,18,19], while three studies employed more than one peptide [14,17,22]. PET/CT images were analyzed qualitatively in one study [18], through both qualitative and semi-quantitative analysis in four studies [19,21,22,23] and semi-quantitatively in the remaining six studies. Regarding qualitative analysis, all the included articles specified the Krenning Score as the selected visual standardized method for assessment, except for two [18,21]. Considering semi-quantitative parameters, the maximum standardized uptake value (SUVmax) was the most common feature measured, followed by SUVmean. SSTR2 was the most common target for IHC (all studies), followed by SSTR5 [14,15,16,18,19,21,22]. The antibodies employed to perform IHC were heterogeneous among the included studies, with monoclonal antibody UMB-1 being the most used to target SSTR2, followed by polyclonal antibodies to target other SSTR subtypes. The scores for the IHC assessment were also heterogeneous, with the semi-quantitative method being the most employed, although with different scores; the DAKO-score for her2-neu and the immunoreactive score (IRS) were the most used (Table 2). Supplemental Table S1 in the Supplemental Materials reports the outcome of each study, reporting both the primary outcome (% concordance) and the outcome from qualitative analysis.

### 3.2. Risk of Bias and Applicability

**Figure 2:**
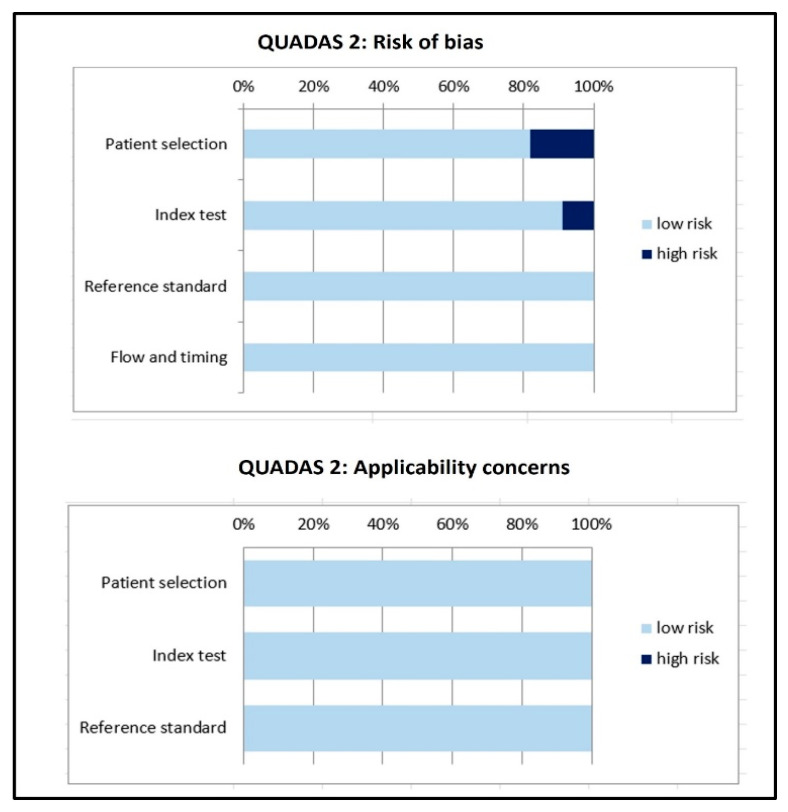
Overall quality assessment according to QUADAS-2 about the risk of bias and applicability concerns of the included studies.

The overall estimation of the risk of bias and concerns regarding the applicability of articles included in the systematic review according to QUADAS-2 are summarized in Figure 2.

### 3.3. Quantitative Synthesis (or Meta-Analysis)

**Figure 3:**
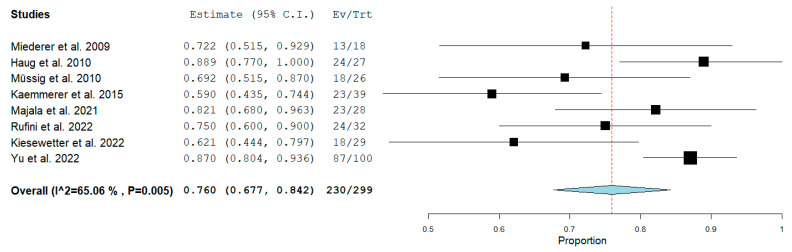
Meta-analysis on the pooled concordance on somatostatin receptor expression between somatostatin receptor PET and immunohistochemistry. For each study and for the pooled value (red line), estimates are reported along with 95% confidence interval values [13,17,18,19,20,21,22,23].

**Figure 4:**
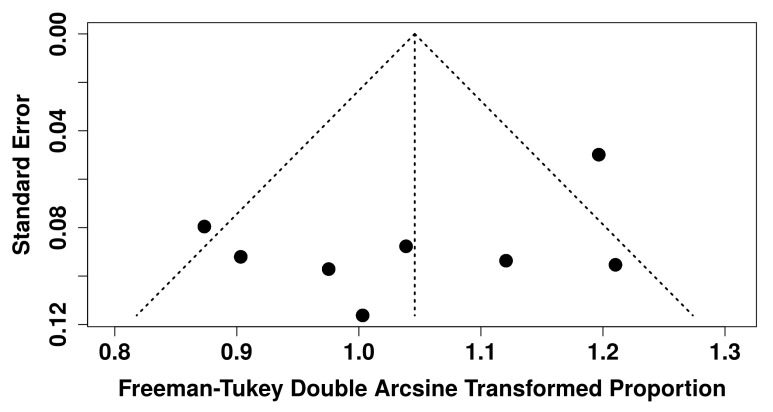
Funnel plot for the meta-analysis. Dots indicate the included studies. The visual analysis of the funnel plot (absence of significant asymmetry in dots distribution) does not indicate a potential publication bias.

Eight studies (299 patients) reporting quantitative data on the concordance between SSTR PET/CT and IHC assessment were included in the quantitative synthesis. The pooled concordance of SSTR expression among SSA-PET/CT and IHC was 76% (95% CI: 67.7–84.2). A moderate heterogeneity was found according to the I^2^ test (65%). Due to the limited number of articles included, there were not sufficient data for significant subgroup analyses further exploring the statistical heterogeneity. No significant publication bias was found according to the visual analysis of the funnel plot and Egger’s test (*p* = 0.18). The forest plot of the meta-analysis is displayed in Figure 3. The funnel plot of the meta-analysis is reported in Figure 4.

## 4. Discussion

This systematic review and meta-analysis were conducted to consolidate evidence on the correlation between the in vitro (IHC) and in vivo (PET/CT) expression of SSTRs in NEN patients, as no extensive analysis of this topic was available in the current literature. Our results highlight the overall good concordance rate (76%) of SSTR expression between in vivo quantification using PET/CT with radiolabeled SSAs and in vitro IHC in NEN samples, both in mixed series of neuroendocrine patients and in selected series of specific NEN diseases such as GEP or lung NEN, which are the predominant types according to the site of origin. Following the study by Miederer and coworkers [20], which was the first group to evaluate the relationship between SUV values from Ga-68-DOTATOC PET/CT and SSTR2 IHC scoring, further studies demonstrated a good correlation between radiolabeled SSA uptake at PET/CT and IHC staining [13,14,15,16,17,19,20,21,22,23].

These results have important clinical implications, as they point out the possibility of using IHC as a surrogate of SSA-PET/CT when this imaging technique is not available. While SSA-PET/CT remains the gold standard for in vivo imaging of SSTR expression, IHC offers a cost-effective, accessible method for guiding the use of SSA in both diagnosis and treatment planning. By assessing the SSTR status via IHC, clinicians can make informed decisions about whether SSAs are a suitable treatment option, particularly in the context of managing functional NETs or planning for peptide receptor radionuclide therapy (PRRT). IHC, which assesses the presence and abundance of SSTRs on NET cells, can help determine if a NET is likely to be responsive to SSA-based therapies, even when PET/CT, which directly visualizes receptor expression, is not accessible [13,14,15,16,17,18,19,20,21,22,23].

Whilst IHC is a helpful tool, there are some inherent limitations, such as sample heterogeneity (tumor tissue is heterogeneous, meaning that SSTR expression may not be uniform across the entire tumor or between metastatic sites; a single biopsy might not fully capture this heterogeneity, unlike SSA-PET/CT, which provides a more comprehensive view of SSTR across the body), sensitivity (IHC might not detect low levels of SSTR expression, whereas SSA-PET/CT can detect even low levels of SSTR expression), and the use of qualitative data (IHC typically provides qualitative information—e.g., presence/absence of SSTR expression—whereas SSA-PET/CT provides quantitative data on receptor density and distribution, which can help in monitoring treatment response) [13,14,15,16,17,18,19,20,21,22,23].

SSA-PET/CT is the most reliable modality for in vivo assessment of SSTR status, providing tumor imaging with high sensitivity. Based on the results of this systematic review and meta-analysis, SSA-PET/CT could be used for restaging during follow-up even in the absence of preoperative or staging imaging study in those cases showing positive (i.e., membranous staining) SSTR2 IHC on tissue samples [20,22]. However, the possibility of a heterogeneous tumor population should be considered as a pattern that can escape the in vitro study, which indicates the presence of SSTR expression in one particular lesion. Moreover, the absence of SSTR2 IHC expression does not exclude positive imaging, therefore, in this case imaging could also be performed; a negative scan will be explained by the in vitro result. In any case, the use of imaging with radiolabeled SSAs maintains its exclusive role in selecting patients for radioligand therapy due to its unique peculiarity of detecting all SSTR-positive lesions.

In the included studies different SSAs were used as a radioligand for imaging, labeled with Gallium-68 in all of the studies. Although the distinct binding affinities for SSTRs (for SSTR2, higher affinity of DOTATATE followed by DOTANOC and then by DOTATOC; for SSTR3, high affinity of DOTANOC only; for SSTR5, high affinity of DOTANOC and DOTATOC), these radiopharmaceuticals have shown comparable results in terms of tumor imaging and ability in detecting SSTR-positive lesions [6]. However, we cannot exclude the possibility of different results of concordance between in vivo and in vitro SSTR expression, taking into account the different SSAs used. For instance, the two studies with the highest concordance percentage used Ga-68-DOTATATE. However, limited available data hampered significant subgroup analyses based on the different SSA used.

Our systematic review and meta-analysis revealed comparable results in terms of agreement between in vivo quantification of SSTR expression and SSTR staining at IHC. Regarding PET image analysis, both qualitative (visual) assessment of the radioligand uptake with adoption of the modified Krenning Score (identical to the original one developed for scintigraphic imaging with In-111 pentetreotide), and semiquantitative analysis with the use of SUV-related measurements were the selected methods for assessment [27]. Despite the many sources of variability influencing the routine use of SUV, including lesion size and uptake time, as well as image reconstruction methods and partial volume effects, SUV analysis was the most frequently used approach, either exclusively or in combination with qualitative analysis. In any case, the choice of the assessment method did not influence the results as a good correlation between the in vivo and the in vitro expression of SSTRs in NEN samples was found regardless of the method used.

For our analysis, we selected only studies using IHC for in vitro evaluation of SSTR expression, a method characterized by widespread use, robustness, and low cost with good correlation with response to somatostatin analogs [28]. Among alternative methods, one is autoradiography, which identifies the receptor protein and, unlike IHC, allows receptor subtype quantification; however, this technique is not widely accessible and requires fresh frozen tissue, a material which is rarely available [29,30]. Another one is RT-PCR, which identifies mRNA encoding SSTR (and not the receptor protein) and allows quantification [31]; however, its disadvantages are the relative complexity of the technology and high costs. When tested, SSTR2 mRNA proved to be a reliable biomarker for predicting PET/CT results, and a good correlation between IHC and RT-PCR for SSTR2 was found [17]. For IHC staining, different scoring systems were applied, reflecting the lack of standardization in this setting. The most used were the DAKO score for Her2-neu and the IRS. The DAKO score is a method initially developed to detect the membrane-bound receptor Her2 in breast carcinoma and considers for scoring only membranous staining [32]; some authors propose this score as the standard method for NENs due to its simplicity [13,17,20]. The IRS was initially developed for IHC detection of estrogen receptors in breast carcinoma [33], evaluating both the intensity of staining and the percentage of positive cells in each staining category. Yu and coworkers [23] compared four different scoring systems and correlated the results with those of Ga-68-DOTATATE PET/CT. Besides the Her2 score and the IRS, they also included the Volante’s score, which was the first method specifically developed for SSTR2 assessment based on the subcellular localization of SSTR and extent of staining, and the H score, which is based on the staining intensity and the percentage of positive cells [12]. These authors showed a significant positive correlation among all methods, as well as with PET/CT imaging results, mainly in samples with homogeneous SSTR2 expressions. However, a lower level of concordance was found when samples with heterogeneous SSTR2 expression were considered (43% of all cases) [23]. Tumor heterogeneity is a notable oncological issue referring to those biological changes occurring within the tumor tissue (intra-tumoral) or among different tumor sites in the same patient (inter-tumoral). For NEN lesions, primary or metastatic, this phenomenon can lead to less-differentiated tumor cells with modifications of SSTR expression and the Ki-67 index, thus influencing tumor behavior in terms of tumor response and patient outcome [34]. In IHC, SSTR and Ki-67 are usually determined on a single lesion without information on the inter-tumoral heterogeneity, a pattern that is well evaluated by PET imaging, a whole-body technique assessing all lesions in the same patient. Moreover, different radiopharmaceuticals are available, which are chosen according to tumor grading, i.e., radiolabeled SSAs for well-differentiated lesions and Fluorine-18 fluorodeoxyglucose (F-18-FDG) for less-differentiated ones. Besides lesion detection, PET/CT with these specific radioligands provides prognostic information [35,36,37,38].

When the tumor grading assessed with IHC was reported, about 11% of patients had lesions classified as high-grade (G3). However, as seven of these studies had been published before the 2017 WHO classification, all high-grade lesions were considered neuroendocrine carcinomas (NECs), although this likely included some NET G3s; in any case, most of these samples had a score 0 or 1 in IHC (only one sample with a score of 3 and a Ki-67 equal to 30% was reported in the study by Miederer and coworkers) [20]. Conversely, in the recent study by Yu and coworkers, most of the high-grade lesions (7 of 10 NEC samples and 9 of 14 NET-G3 samples) had a positive SSTR2 IHC. This finding fits well with the well-known and relatively unexpected expression of SSTRs in NECs (poorly differentiated by definition) found in up to 25% of NECs [39]. The widespread use of the more reliable and sensitive IHC tool (the UMB-1 monoclonal antibody) may also explain the more recent data on SSTRs IHCs in high-grade NENs [28].

For IHC staining the most frequently used target was SSTR2, which is the subtype receptor well recognized by all three radioligands for PET/CT. Except for the study by Majala and coworkers [19], who found no association between SSTR2 expression and SUVmax, in the other studies SSTR2 IHC scores and SUV values behaved in the same way, as negative SSTR2 expressions in IHC matched low SUV values, whereas positive receptor expression matched high SUV values, indicating that SUV values can be used as a parameter for SSTR2 density [13,20,22,23]. However, discordant results were reported in more than one study, mostly regarding SSTR2-negative tumors showing ^68^Ga-SSA uptake [13,22,23]. Besides biopsy sampling errors or tissue handling with improper receptor antigen preservation and poor IHC results, one of the main challenges to obtain absolute correlation between the in vivo expression at PET/CT and SSTR IHC remains the heterogeneity in tumor receptor expression. These data suggest the need for larger studies to address discrepancies between imaging results and IHC. Moreover, automated analysis of IHC samples may help in reducing inter-observer variability by making IHC more reproducible [14].

Most of the studies selected, besides SSTR2, evaluated the expression of other subtype receptors in IHC with less defined and less concordant results with respect to SSTR2 (Table 2).

This systematic review and meta-analysis acknowledges the following limitations which may introduce a potential bias that should not be overlooked: (i) the included studies were mainly retrospective, with only one prospective study; (ii) the majority of the studies were monocentric, implying site-specific potential biases; (iii) the included studies were heterogeneous under several aspects such as in the specific number of patients (variable from 18 to 95), the NET site (GEP, lung, others), the tumor sample type (biopsy vs. surgical procedure, with a few studies not detailing this aspect), the type of radiolabeled peptide used for PET/CT (albeit always somatostatin analogs), its injected activity and uptake time, IHC targets (mainly SSTR2, but in some cases also other SSTR subtypes), and IHC assessment methods. Unfortunately, due to the limited number of articles included in our meta-analysis there were not sufficient data for significant subgroup analyses further exploring the statistical heterogeneity.

Suggestions for further studies about the correlation between in vivo and in vitro SSTR expression in NENs include the design of large prospective and multicentric trials stratified by tumor site and grade; analyses of correlation with the standardization of IHC scoring; and trials evaluating possible differences in this correlation taking into account the different PET radiopharmaceuticals used.

## 5. Conclusions

In conclusion, this systematic review and meta-analysis confirm a good concordance rate between SSTR expressions assessed in vivo with SSA-PET/CT and in vitro with IHC. These findings highlight the potential of integrating IHC into clinical decision-making for NET management. Original studies with larger cohorts of patients, homogeneous NET type, and a prospective and multicentric nature are warranted to further strengthen these results, also considering the importance of assessing SSTR expression in patients who need radioligand therapy. Indeed, current scientific research in this field is expanding the horizons of therapy in NEN patients, focusing on the therapeutic outcomes of the known SSAs labeled with alpha-emitting (such as Actinium-225, Astatine-211, and others) or beta-emitting nuclides with Auger emissions (e.g., Terbium-161), or studying the safety and outcomes of radiolabeled SSR antagonists. The common basis for all these therapeutic opportunities is and will be the proper assessment of SSTR expression both in vivo with SSA-PET/CT and in vitro with IHC.

## Figures and Tables

**Table 1:** General study information and patients’ key characteristics.

First Author and Year	Country	Journal	Study Design	Funding Sources	No. Patients	M/F	Age, Years (Range)	NEN Site	Specimen Type
Miederer M 2009 [20]	Germany	EJNMMI	Retrospective	N.R.	18	10/8	53 [mean]	GEP: 11; L: 1; others: 6	Biopsy: 4; surgery: 14
Haug AR 2010 [13]	Germany	Radiologe	Retrospective	N.R.	27	18/9	59.6 [mean] (40–73)	GEP: 19; L: 3; others: 5	N.R.
Müssig K 2010 [21]	Germany	Horm Metab Res	Retrospective	N.R.	36	19/17	58 [mean] SD 12	GEP: 23; L: 1; others: 12	Biopsy: 17; surgery: 23 (2 tumors in 4 patients)
Kaemmerer D 2011 [16]	Germany	EJNMMI	Retrospective	N.R.	17	N.R.	58 [median] (33–82)	GEP: 17	N.R.
Kaemmerer D 2012 [15]	Germany	Int J Clin Exp Patho	Retrospective	N.R.	21	N.R.	56 [median] SD 11 (33–76)	GEP: 21	N.R.
Kaemmerer D 2014 [14]	Germany	Int J Clin Exp Patho	Retrospective	N.R.	25	15/10	(33–82)	GEP: 25	Surgery: 31
Kaemmerer D 2015 [17]	Germany	Pancreas	Retrospective	N.R.	19	11/8	(33–77)	panNEN: 19	Biopsy: 39 (primary tumor and metastases)
Majala S 2021 [19]	Finland	Cancers	Prospective	Yes	21	13/8	54.9 [mean] SD 18.1	panNEN: 21	Biopsy: 1; surgery: 20
Rufini V 2022 [22]	Italy	EJNMMI	Retrospective	Yes	32	20/12	62 [median] (29–82)	L: 32	Biopsy: 8; surgery: 24
Kiesewetter B 2022 [18]	Austria	ESMO Open	Retrospective	No	34	12/22	78 [median] (28–88)	L: 34	N.R.
Yu J 2022 [23]	China	Neuroendocrinology	Retrospective	Yes	95	54/41	N.R.	GEP: 95	Biopsy: 61; surgery: 39

N.R.: not reported; GEP: gastroenteropancreatic; L: lung; panNEN: pancreas NEN.

**Table 2:** Key characteristics about PET and immunohistochemistry.

First Author and Year	Hybrid Imaging	Tomograph	Peptide	Injected Activity (MBq)	Uptake Time (min)	Image Analysis	Semi-quantitative Parameters	IHC Targets	IHC Antibodies (Ab)	IHC Assessment
Miederer M 2009 [20]	PET/CT	Siemens Biograph 16	DOTA-TOC	112 [mean], SD 15	20	Semi-quantitative	SUVmean, SUVmax	SSTR2	Antibodies against SSTR2 n.b.s.	Semi-quantitative (0–3) [DAKO-score for her2-neu]
Haug AR 2010 [13]	PET/CT	Philips Gemini	DOTA-TATE	200 [fixed dose]	60	Semi-quantitative	SUVmax	SSTR2	Antibodies against SSTR2 n.b.s.	Semi-quantitative (0–3) [DAKO-score for her2-neu]
Müssig K 2010 [21]	PET/CT	Siemens Biograph 16	DOTA-TOC	150 [mean]	20	Qualitative + semi-quantitative	SUVmax	SSTR2, SSTR3, SSTR5	Monoclonal Ab (SSTR2), policlonal Ab (SSTR3, SSTR5)	(1) Semi-quantitative (0–3). (2) Qualitative (+ weak, ++ moderate, +++ strong)
Kaemmerer D 2011 [16]	PET/CT	N.R.	DOTA-NOC	N.R.	N.R.	Semi-quantitative	SUVmean, SUVmax	SSTR2A, SSTR1, SSTR3-5	Monoclonal Ab UMB-1 (SSTR2A), policlonal Ab (SSTR1, SSTR3-5)	Semi-quantitative (IRSmod 0–12 and IRS 0–3)
Kaemmerer D 2012 [15]	PET/CT	N.R.	DOTA-NOC	N.R.	N.R.	Semi-quantitative	SUVmax	SSTR2A, SSTR1, SSTR3-5	Monoclonal Ab UMB-1 (SSTR2A), policlonal Ab (SSTR1, SSTR3-5)	Two semi-quantitative scores: IRS (IRSmod 0–12 and IRS 0–3) and Her2 score (0–3)
Kaemmerer D 2014 [14]	PET/CT	Siemens Biograph Duo	DOTA-NOC, DOTA-TATE	N.R.	N.R.	Semi-quantitative	SUVmean, SUVmax	SSTR2A, SSTR1, SSTR4, SSTR5	Monoclonal Ab (SSTR2A), policlonal Ab (SSTR1, SSTR4, SSTR5)	Two semi-quantitative scores: IRS (IRSmod 0–12) and Her2 score (0–3)
Kaemmerer D 2015 [17]	PET/CT	Siemens Biograph Duo	DOTA-TATE, DOTA-TOC, DOTA-NOC	122.4 [mean], SD 13.98 (range 86–149)	60	Semi-quantitative	SUVmean, SUVmax	SSTR2A	Monoclonal Ab UMB-1 (SSTR2A)	Semi-quantitative. (1) IRS. (2) DAKO-score for her2-neu (0–3)
Majala S 2021 [19]	PET/CT	GE Discovery; Siemens Biograph mCT; Philips Gemini; Siemens Biograph 6	DOTA-NOC	142.7 [mean], SD 18.9	54 ± 9	Qualitative + Krenning Score (0–4) + semi-quantitative	SUVmax	SSTR1–5	Monoclonal Ab (UMB-1 for SSTR2)	Semi-quantitative (Overall score by Elston and Korner score, 0–3)
Rufini V 2022 [22]	PET/CT	Philips Gemini GXL, Siemens Biograph mCT	DOTA-TOC, DOTA-NOC	2 MBq/kg	45 ± 10	Qualitative + Krenning Score (0–4) + semi-quantitative	SUVmax	SSTR2, SSTR5	Monoclonal Ab (UMB-1 for both)	Semi-quantitative (Volante score 0–3)
Kiesewetter B 2022 [18]	PET/CT	Siemens Biograph 64	DOTA-NOC	(range 160–180)	45–60	Qualitative	/	SSTR2, SSTR5	Abcam for both n.b.s.	Qualitative (0, +, ++, +++) and semi-quantitative (0–300)
Yu J 2022 [23]	PET/CT	Siemens Biograph 64	DOTA-TATE	(range 100–200)	60	Qualitative + Krenning Score (0–4) + semi-quantitative	SUVmax	SSTR2	Monoclonal Ab	Four semi-quantitative scores: (1) Her2 score (0–3) (2) Volante score (0–3) (3) H score (0–300) (4) IRSmod (0–12)

n.b.s.: not better specified; N.R.: not reported; IRS: immunoreactive score.

## Data Availability

No new data were created for this manuscript.

## Supplementary Materials

The following supporting information can be downloaded at: https://www.mdpi.com/article/10.3390/ijms26146551/s1.
