# Supplementary material for: In Vivo Versus In Vitro Somatostatin Receptor Expression in Neuroendocrine Neoplasms: A Systematic Review and Meta-Analysis of Correlation Studies
Source: Int J Mol Sci. 2025 Jul 8;26(14):6551. doi: 10.3390/ijms26146551 (PMC12294157; doi:10.3390/ijms26146551)
Supplement: Supplementary file 1 [file ijms-26-06551-s001.zip › ijms-3714212-supplementary.pdf]

Supplemental material: PRISMA Checklist and Table on study outcomes

Section and Topic	Item #	Checklist item	Location where item is reported
**TITLE**			
Title	1	Identify the report as a systematic review.	Done in the title
**ABSTRACT**			
Abstract	2	See the PRISMA 2020 for Abstracts checklist.	Done
**INTRODUCTION**			
Rationale	3	Describe the rationale for the review in the context of existing knowledge.	Done in the introduction
Objectives	4	Provide an explicit statement of the objective(s) or question(s) the review addresses.	Done in the introduction
**METHODS**			
Eligibility criteria	5	Specify the inclusion and exclusion criteria for the review and how studies were grouped for the syntheses.	Done in the methods
Information sources	6	Specify all databases, registers, websites, organisations, reference lists and other sources searched or consulted to identify studies. Specify the date when each source was last searched or consulted.	Done in the methods
Search strategy	7	Present the full search strategies for all databases, registers and websites, including any filters and limits used.	Done in the methods
Selection process	8	Specify the methods used to decide whether a study met the inclusion criteria of the review, including how many reviewers screened each record and each report retrieved, whether they worked independently, and if applicable, details of automation tools used in the process.	Done in the methods
Data collection process	9	Specify the methods used to collect data from reports, including how many reviewers collected data from each report, whether they worked independently, any processes for obtaining or confirming data from study investigators, and if applicable, details of automation tools used in the process.	Done in the methods
Data items	10a	List and define all outcomes for which data were sought. Specify whether all results that were compatible with each outcome domain in each study were sought (e.g. for all measures, time points, analyses), and if not, the methods used to decide which results to collect.	Done in the methods
	10b	List and define all other variables for which data were sought (e.g. participant and intervention characteristics, funding sources). Describe any assumptions made about any missing or unclear information.	Done in the methods
Study risk of bias assessment	11	Specify the methods used to assess risk of bias in the included studies, including details of the tool(s) used, how many reviewers assessed each study and whether they worked independently, and if applicable, details of automation tools used in the process.	Done in the methods
Effect measures	12	Specify for each outcome the effect measure(s) (e.g. risk ratio, mean difference) used in the synthesis or presentation of results.	Done in the methods
Synthesis methods	13a	Describe the processes used to decide which studies were eligible for each synthesis (e.g. tabulating the study intervention characteristics and comparing against the planned groups for each synthesis (item #5)).	Done in the methods
	13b	Describe any methods required to prepare the data for presentation or synthesis, such as handling of missing summary statistics, or data conversions.	Done in the methods
	13c	Describe any methods used to tabulate or visually display results of individual studies and syntheses.	Done in the methods
	13d	Describe any methods used to synthesize results and provide a rationale for the choice(s). If meta-analysis was performed, describe the model(s), method(s) to identify the presence and extent of statistical heterogeneity, and software package(s) used.	Done in the methods

Supplemental material: PRISMA Checklist and Table on study outcomes

Section and Topic	Item #	Checklist item	Location where item is reported
	13e	Describe any methods used to explore possible causes of heterogeneity among study results (e.g. subgroup analysis, meta-regression).	Done in the methods
	13f	Describe any sensitivity analyses conducted to assess robustness of the synthesized results.	Not applicable
Reporting bias assessment	14	Describe any methods used to assess risk of bias due to missing results in a synthesis (arising from reporting biases).	Done in the methods
Certainty assessment	15	Describe any methods used to assess certainty (or confidence) in the body of evidence for an outcome.	Not applicable
**RESULTS**			
Study selection	16a	Describe the results of the search and selection process, from the number of records identified in the search to the number of studies included in the review, ideally using a flow diagram.	Done in the results
	16b	Cite studies that might appear to meet the inclusion criteria, but which were excluded, and explain why they were excluded.	Done in the results
Study characteristics	17	Cite each included study and present its characteristics.	Done in the results
Risk of bias in studies	18	Present assessments of risk of bias for each included study.	Done in the results
Results of individual studies	19	For all outcomes, present, for each study: (a) summary statistics for each group (where appropriate) and (b) an effect estimate and its precision (e.g. confidence/credible interval), ideally using structured tables or plots.	Done in the results
Results of syntheses	20a	For each synthesis, briefly summarise the characteristics and risk of bias among contributing studies.	Done in the results
	20b	Present results of all statistical syntheses conducted. If meta-analysis was done, present for each the summary estimate and its precision (e.g. confidence/credible interval) and measures of statistical heterogeneity. If comparing groups, describe the direction of the effect.	Done in the results
	20c	Present results of all investigations of possible causes of heterogeneity among study results.	Done in the results
	20d	Present results of all sensitivity analyses conducted to assess the robustness of the synthesized results.	Not applicable
Reporting biases	21	Present assessments of risk of bias due to missing results (arising from reporting biases) for each synthesis assessed.	Not applicable
Certainty of evidence	22	Present assessments of certainty (or confidence) in the body of evidence for each outcome assessed.	Not applicable
**DISCUSSION**			
Discussion	23a	Provide a general interpretation of the results in the context of other evidence.	Done in the discussion
	23b	Discuss any limitations of the evidence included in the review.	Done in the discussion
	23c	Discuss any limitations of the review processes used.	Done in the discussion

Supplemental material: PRISMA Checklist and Table on study outcomes

Section and Topic	Item #	Checklist item	Location where item is reported
	23d	Discuss implications of the results for practice, policy, and future research.	Done in the discussion
**OTHER INFORMATION**			
Registration and protocol	24a	Provide registration information for the review, including register name and registration number, or state that the review was not registered.	Stated that the protocol was not registered
	24b	Indicate where the review protocol can be accessed, or state that a protocol was not prepared.	Stated in the methods
	24c	Describe and explain any amendments to information provided at registration or in the protocol.	Not applicable
Support	25	Describe sources of financial or non-financial support for the review, and the role of the funders or sponsors in the review.	Done
Competing interests	26	Declare any competing interests of review authors.	Done
Availability of data, code and other materials	27	Report which of the following are publicly available and where they can be found: template data collection forms; data extracted from included studies; data used for all analyses; analytic code; any other materials used in the review.	Done

From: Page MJ, McKenzie JE, Bossuyt PM, Boutron I, Hoffmann TC, Mulrow CD, et al. The PRISMA 2020 statement: an updated guideline for reporting systematic reviews. BMJ 2021;372:n71. doi: 10.1136/bmj.n71.
This work is licensed under CC BY 4.0. To view a copy of this license, visit https://creativecommons.org/licenses/by/4.0/

Supplemental material: PRISMA Checklist and Table on study outcomes

Supplemental Table S1. Study outcomes.

First Author & Year	Primary Outcome (% Concordance)	Discussion Notes	Outcomes for Qualitative Analysis
Miederer M 2009 *	13/18 = 72.2% ^	- SSTR-IHC score 0: SUV<10; SSTR-IHC score 2 and 3: SUV>15.	/
Haug AR 2010 *	24/27 = 88.8% ^	/	/
Müssig K 2010 *	18/26 = 69.2% ^	- SSTR3 and SSTR5 IHC was not associated with tracer uptake.	- 14 tumors were also scanned with [^{111}In]In-DTPAOC scintigraphy (significant association between qualitative IHC SSTR2 detection and positive imaging)
Kaemmerer D 2011	- Highly significant correlation between IRS of SSTR2A and SUVmax ($p < 0.001$).<br>- Significant correlation between IRS of SSTR2A and SUVmean ($p < 0.01$).	/	- Highly significant correlation between IRS of SSTR5 and SUVmax ($p < 0.04$).
Kaemmerer D 2012	- Significant correlation between IRS of SSTR2A and SUVmax (0.33; $p = 0.05$).<br>- Positive correlation with strong trend between Her2-score of SSTR2A and SUVmax (0.33; $p = 0.08$).	/	- The IRS-classification and the Her2-score were found to be statistically comparable, and their correlation was highly significant for each SSTR assessment ($p < 0.01$).
Kaemmerer D 2014	- Significant correlation between Her2 score of SSTR2A and SUVmax ($p = 0.028$) and SUVmean ($p = 0.001$).<br>- SUVmax was calculated in 28/31 lesions; SUVmean was calculated in 27/31 lesions.	- No significant correlation to SUVmax and SUVmean for SSTR2A by IRS score, SSTR1 and SSTR4-5 status.	- A virtual IHC score named "BB1" from digitalized slices was created to compare the manual and automated analysis of SSTR expression.<br>- BB1 score showed a significant correlation: with the IRS and Her2-score of SSTR2A and SSTR5; with the Her2-score of SSTR4.<br>- BB1 score of SSTR2A also significantly correlated with SUVmax and SUVmean.
Kaemmerer D 2015 *	23/39 = 58.9% (for SUVmax) ^	/	- RT-qPCR for SSTR2 mRNA expression (10 specimens): very good correlation between SUVmean and mRNA ($p < 0.001$); good correlation between SUVmax and mRNA ($p = 0.04$). Strong trend towards an association between IRS score of SSTR2A and mRNA ($p = 0.07$); no correlation between Her2/neu score of SSTR2A and mRNA ($p = 0.25$).<br>- No differences between SUVmax and SUVmean among primary and secondary lesions; there were differences in SSTR2A expression between samples from primary and secondary lesions.

Supplemental material: PRISMA Checklist and Table on study outcomes

Majala S 2021 *	23/28 = 82.1% ^	- When PET/CT was scored either positive or negative (visual), all DOTANOC-avid tumors (n = 22/23) expressed membranous SSTR2, and the only DOTANOC-negative tumor did not express membranous SSTR2 (n = 1/23) ($p = 0.043$).<br>- There was no association between SSTR2 expression and SUVmax.	- There was no correlation between DOTA-NOC PET/CT and SSTR1 or SSTR3-5 IHC expression.<br>- SSTR5 expression was higher in tumors with low Ki-67.<br>- [^{18}F]FDG PET/CT was also performed: 59% tumors were [^{18}F]FDG-negative; there was a negative correlation between [^{18}F]FDG uptake and SSTR5 membranous expression; there was no correlation between [^{18}F]FDG PET/CT and IHC SSTR1-4 expression.
Rufini V 2022 *	24/32 = 75% ($p = 0.003$)	- A significant correlation between SUVmax at SSTR-PET/CT and SSTR2-IHC scores was found, with low SUVmax corresponding to negative IHC and higher SUVmax to positive IHC ($p = 0.002$).	- In 14/32 patients [^{18}F]FDG PET/CT was also performed (positive in 71.4% of cases).<br>- [^{18}F]FDG PET/CT strongly correlated with Ki-67 in atypical carcinoids ($p < 0.0001$).
Kiesewetter B 2022 *	18/29 = 62%	/	/
Yu J 2022 *	87/100 = 87% (Her2-score);<br>82/100 = 82% (Volante score);<br>87/100 = 87% (H score);<br>79/100 = 79% (IRSmod)	/	- In 57% of cases, the 4 scoring systems were highly concordant with each other ($p < 0.001$) and highly correlated with DOTA-TATE PET/CT results ($p < 0.001$).

* = these articles provided sufficient data for the meta-analysis; ^ = calculated by the reviewers.
